# Pre-Treatment Whole Blood Gene Expression Is Associated with 14-Week Response Assessed by Dynamic Contrast Enhanced Magnetic Resonance Imaging in Infliximab-Treated Rheumatoid Arthritis Patients

**DOI:** 10.1371/journal.pone.0113937

**Published:** 2014-12-12

**Authors:** Kenzie D. MacIsaac, Richard Baumgartner, Jia Kang, Andrey Loboda, Charles Peterfy, Julie DiCarlo, Jonathan Riek, Chan Beals

**Affiliations:** 1 Merck & Co. Inc., Department of Genetics and Pharmacogenomics, Boston, Massachusetts, United States of America; 2 Merck & Co. Inc., Department of Biometrics Research, Whitehouse Station, New Jersey, United States of America; 3 Spire Sciences Inc., Boca Raton, Florida, United States of America; 4 Virtual Scopics, Rochester, New York, United States of America; 5 Merck & Co. Inc., Clinical Research, Whitehouse Station, New Jersey, United States of America; Center for Rheumatic Diseases, India

## Abstract

Approximately 30% of rheumatoid arthritis patients achieve inadequate response to anti-TNF biologics. Attempts to identify molecular biomarkers predicting response have met with mixed success. This may be attributable, in part, to the variable and subjective disease assessment endpoints with large placebo effects typically used to classify patient response. Sixty-one patients with active RA despite methotrexate treatment, and with MRI-documented synovitis, were randomized to receive infliximab or placebo. Blood was collected at baseline and genome-wide transcription in whole blood was measured using microarrays. The primary endpoint in this study was determined by measuring the transfer rate constant (K_trans_) of a gadolinium-based contrast agent from plasma to synovium using MRI. Secondary endpoints included repeated clinical assessments with DAS28(CRP), and assessments of osteitis and synovitis by the RAMRIS method. Infliximab showed greater decrease from baseline in DCE-MRI K_trans_ of wrist and MCP at all visits compared with placebo (*P*<0.001). Statistical analysis was performed to identify genes associated with treatment-specific 14-week change in K_trans_. The 256 genes identified were used to derive a gene signature score by averaging their log expression within each patient. The resulting score correlated with improvement of K_trans_ in infliximab-treated patients and with deterioration of K_trans_ in placebo-treated subjects. Poor responders showed high expression of activated B-cell genes whereas good responders exhibited a gene expression pattern consistent with mobilization of neutrophils and monocytes and high levels of reticulated platelets. This gene signature was significantly associated with clinical response in two previously published whole blood gene expression studies using anti-TNF therapies. These data provide support for the hypothesis that anti-TNF inadequate responders comprise a distinct molecular subtype of RA characterized by differences in pre-treatment blood mRNA expression. They also highlight the importance of placebo controls and robust, objective endpoints in biomarker discovery.

***Trial Registration:*** ClinicalTrials.gov NCT01313520

## Introduction

Anti-TNF biologics are an important class of therapeutics in the treatment of rheumatoid arthritis, but unfortunately approximately 30% of patients achieve inadequate response. Variability in response is incompletely understood. It has been linked to smoking status, concomitant treatment with methotrexate (MTX) and other DMARDs, disease severity, and patient disability [1]. Hypothesizing that inadequate responders constitute a distinct molecular subtype, several blood gene expression studies have been undertaken to identify gene expression-based biomarkers predicting response to anti-TNF [2]–[9]. Such gene signatures consist of characteristic patterns of mRNA expression distinguishing responders and non-responders. A recent study attempted to replicate the reported association of eight pre-specified signatures with response status and reported that a single signature was validated with modest predictive value [10]. The generally poor validation of published signatures is perhaps not surprising since the signatures tested were not derived from consistent patient populations or blood cell fractions. In addition, in these studies response was assessed using composite disease activity scores like the DAS28 or American College of Rheumatology (ACR) response criteria. Since these endpoints are known to be subject to large placebo effects [11], inclusion of appropriate placebo controls may be particularly crucial for successful biomarker discovery. Unfortunately none of these studies included a placebo control, confounding true responders and patients with flaring disease that subsequently subsides in a treatment-independent manner.

Given the limitations of the DAS28 and similar measures, biomarker discovery using objective disease assessments, like magnetic resonance imaging (MRI), is attractive. Uniquely, MRI is able to evaluate the inflammation of synovium and bone, which are thought to ultimately result in articular cartilage loss and bone erosion, respectively. It is most frequently monitored using the RAMRIS method [12], a semi-quantitative scoring system where bone erosion, osteitis, and synovitis are evaluated by MRI. Dynamic contrast enhanced MRI (DCE-MRI), is an alternative quantitative method to measure synovitis by administering gadolinium-based contrast agents (GBCA) intravenously and collecting sequential images of the joint in a time course [13]. The enhancement curve generated by DCE-MRI can be used to estimate physiological parameters, such as K_trans_, the volume transfer constant of GBCA between blood plasma and the synovium. This endpoint is related to capillary permeability and vascularity in the synovium, and correlates strongly with histological measures of inflammation [14]. Both RAMRIS and DCE-MRI are valid measures for detecting treatment effect, but they are not interchangeable and may reflect somewhat different biological processes related to joint inflammation.

In the present study, we measured pre-treatment gene expression in whole blood and employed DCE-MRI of the wrist to monitor disease progression in a randomized, controlled, multi-site trial of infliximab plus MTX versus placebo plus MTX. Analysis of these data identified a 256 gene signature associated with disease activity measured by K_trans_.

## Methods

This analysis was conducted as part of a 14-week, randomized, double-blind, placebo-controlled, methodology study (Study Protocol PO8136, ClinicalTrials.gov registration: NCT01313520) conducted from April 6, 2011 to March 29, 2012 in 4 clinical centers in Europe. The study was conducted in accordance with principles of Good Clinical Practice and was approved by the National Ethics Committee in Romania and the National Ethics Committee, Clinical Research of Drugs and Methods of Treatment in Moldova. All subjects provided informed written consent. Details of the study methods are provided as a supplement and have been submitted elsewhere for publication.

### Subjects

Participants had a diagnosis of RA for at least 6 months (based on the American College of Rheumatology (ACR) 1987 criteria), at least 6 tender and 6 swollen joints (using the 28 joint set), C-reactive protein (CRP) ≥1.0 mg/L, and were on a stable dose of methotrexate. Subjects who were on stable doses (<10mg/day) of prednisone or non-steroidal anti-inflammatory drugs (NSAIDs) maintained those treatments through the study. All subjects were naïve to anti-TNF biologics, and were required to have RAMRIS synovitis score ≥1 in the radio-carpal or intercarpal joints of one hand based on centralized expert assessment.

### Treatment

At weeks 0, 2, 6, and 14, participants received infliximab 3 mg/kg in 0.9% NaCl or 0.9% NaCl alone. Patients continued to receive their standard dose and regimen of disease modifying antirheumatic drugs (DMARDS).

### Clinical assessments

DAS28(CRP) is a composite score of the number of tender joints (28 joint count), the number of swollen joints (28 joint count), patient global assessment of disease (GADP) on a 100 mm visual analog scale (VAS), and CRP (mg/dL) [15].

### MRI assessments

MRI of the most clinically involved hand and wrist was acquired at baseline and weeks 2, 4, and 14 in order to measure K_trans_. Additionally, synovitis, osteitis and bone erosion were scored using the Outcome Measures in Rheumatology (OMERACT) RA MRI Score (RAMRIS) method [16] at baseline, 2, 4 and 14 weeks by two independent radiologists blinded to visit order and treatment assignments. Details are provided in the supplemental methods.

### Statistical methods

K_trans_ of wrist synovium was the primary endpoint of the study. Secondary endpoints included K_trans_ of metacarpophalangeal joints (MCP), DAS28(CRP), and RAMRIS scores. The DCE-MRI endpoints and the continuous clinical endpoints were compared between treatment groups using constrained Longitudinal Data Analysis (cLDA) proposed by Liang and Zeger [17]. The mean change from baseline to a given time point was estimated and tested from this model. A log transformation of the DCE-MRI parameters was performed to better meet the assumption of normality that is necessary for the cLDA. For RAMRIS, the average of the two readers' scores was used (except in one case for which scores from only one reader were available), and statistical significance was determined using non-parametric van Elteren and Wilcoxon rank sum tests.

### RNA extraction and microarray data generation

RNA was isolated from PAXgene blood samples (2.5ml) according to the manufacturer's instructions. Isolated total RNA samples were assayed for quality (Agilent Bioanalyzer) and yield (Ribogreen) metrics prior to amplification. Fifty-nine samples passing quality control (QC) were then amplified using the NuGEN Ovation WB protocol and hybridized to Rosetta/Merck Human RSTA Custom Affymetrix 2.0 microarrays. Microarray data have been deposited in the Gene Expression Omnibus archive (GEO accession GSE58795).

### Microarray QC and normalization

QC analysis was performed in the absence of treatment allocation information. QC metrics, including average background signal, scale factor, beta-actin and GAPDH 3' to 5' ratios, and the number of genes called 'present' using the MAS5 algorithm, were examined to assess potential technical quality issues related to amplification and RNA degradation. No problematic samples were identified. Array probe intensity normalization was performed using the RMA algorithm in R/Bioconductor. Gene expression patterns and their principal components of variation were checked against the principal components of sample quality and process parameters (QC). The first two QC principal components were observed to correlate significantly with several principal components of variation in the expression data. Therefore, prior to further analysis, the RMA-normalized expression data was detrended by regressing the log2 intensity of each probe set against the first two QC principal components.

### Gene Expression analysis

Detectably expressed probe sets were defined as those with a MAS5 ‘present’ call in at least 25% of all samples. Log expression values of probe sets mapping to the same gene were averaged. Signature scores were used to summarize the expression of the genes comprising pre-specified signatures and were calculated by averaging the log expression intensity values for each gene within a subject. Since signatures may comprise genes both correlated and anti-correlated with treatment response, the mean log expression values for anti-correlated genes was multiplied by -1 to preserve the effect of directionality.

To identify genes whose baseline expression correlated with treatment effect, we modeled the 14-week change in each disease activity endpoint as a function of treatment and gene expression at baseline. For each detectably expressed gene, we performed ordinary least squares regression with terms for log expression intensity, treatment allocation, their interaction, and baseline disease activity. Q-Q plots of interaction term p-values vs. the uniform distribution were generated for DAS28, RAMRIS osteitis, RAMRIS synovitis, and K_trans_ to identify deviation from random expectation. For K_trans_, we defined a 256 gene signature associated with 14-week treatment response by identifying genes with a nominally significant gene expression by treatment interaction term (p<0.01). The Pearson correlation matrix for all 256 genes in the predictive signature was calculated and used to cluster the genes into four groups. Details on clustering are provided in the supplemental methods.

The value of gene expression for predicting 14-week change in disease activity was assessed using a random subsampling strategy. In each round of sampling a training set of 40 subjects was randomly selected, and genes with a significant (p<0.01) expression by treatment interaction term were identified in this training set. Expression of these genes was averaged into a score for each subject. Using only the subjects in the training set, we then fit two linear models of disease activity: the first included terms for baseline disease activity, treatment, signature score, and signature score by treatment interaction, the second included only terms for treatment and baseline disease activity. Each of these models was then used to predict 14-week change in disease activity for the held-out test subjects, and the resulting mean squared prediction errors over ten rounds of sampling were compared.

To evaluate the statistical association between signature score and other disease activity endpoints, we fit a linear model of change from baseline data including terms for baseline disease activity, treatment allocation, signature score and the signature by treatment interaction, and examined the effect size and p-value associated with the gene signature main effect and interaction term.

## Results

Sixty-one adults with moderate to severe RA were enrolled into the study. The cohort was 92% female with a mean age (SD) of 50 (10) years. Ninety-one percent were positive for Rheumatoid Factor. Baseline characteristics are described in Table 1. The mean baseline DAS28(CRP) score was 6.2, indicating high disease activity. Baseline MRI scores also were consistent with high disease activity. There were no significant differences in any of these characteristics, or in prednisone or NSAID use, between the two arms, although higher RAMRIS-synovitis scores in the infliximab group approached significance (*P* = 0.058).

**Table 1:** Baseline characteristics.

	Infliximab (n = 30)	Placebo (n = 31)	Total (n = 61)
Age. Years, mean (SD)	50 (10)	50 (11)	50 (10)
Gender, female, n (%)	28 (93)	27 (90)	56 (92)
DAS28(CRP), mean (SD)	6.1 (0.7)	6.2 (0.7)	6.2 (0.7)
Number of Tender Joints, mean (SD)	19.5 (4.9)	20.1 (5.1)	19.8 (4.9)
Number of Swollen Joints, mean (SD)	12.5 (3.8)	12.4 (3.1)	12.4 (3.4)
CRP (mg/L), median (quartiles)	9.2 (3.4–24.6)	9.1 (5.1–24.6)	9.3 (3.9–25.7)
Rheumatoid Factor Positive, n (%)	26 (86.7)	28 (96.5)	54 (91.5)
K_trans_ wrist enhancing synovium, mean (SD)	0.035 (0.01)	0.034 (0.01)	0.034 (0.01)
K_trans_ MCP enhancing synovium, mean (SD)	0.034 (0.03)	0.031 (0.01)	0.032 (0.02)
K_trans_ enhancing tissue, mean (SD)	0.027 (0.01)	0.025 (0.01)	0.026 (0.01)
RAMRIS synovitis, mean (SD)	11.08 (5.14)	8.27 (4.56)	9.66 (5.02)
RAMRIS osteitis, mean (SD)	9.70 (10.15)	11.2 (11.44)	10.45 (10.75)

One participant meeting clinical criteria did not have sufficient synovitis at baseline to qualify, but was randomized to treatment in error. Since the patient was eligible on clinical grounds, the patient was not discontinued, and was included in all analyses. All subjects completed the study.

### MRI outcomes

Mean K_trans_ of synovium in the wrist derived from DCE-MRI analysis showed a significant treatment effect as early as 2 weeks following initiation of infliximab. This treatment effect was observed at each subsequent time point as well (Table 2). Placebo treatment resulted in no change in K_trans_ of wrist or MCP synovium. Similar treatment effects were observed on mean K_trans_ of total enhancing tissue (synovitis and osteitis) in the wrist and MCPs (data not shown).

**Table 2:** MRI and clinical endpoints.

Clinical endpoint	Placebo (n = 31)			Infliximab (n = 30)		
	2W	4W	14W	2W	4W	14W
Log K_trans_ Wrist	0.11 (0.29)	0.07 (0.34)	0.13 (0.37)	−0.15 (0.36)**	−0.20 (0.25)***	−0.29 (0.49)***
DAS28 (CRP)	−0.21 (0.58)*	−0.48 (0.67)***	−0.82 (0.83)***	−1.02 (0.75)***	−1.29 (0.80)***	−1.80 (1.19)***
RAMRIS synovitis	−0.03 (0.4)	0.03 (0.4)	0.09 (1.08)	−0.46 (0.84)*	−0.6 (0.94)**	−0.75 (1.56)*
RAMRIS osteitis	0.33 (0.93)	0.62 (1.35)	0.56(3.02)	−0.88 (2.21)***	−1.05 (2.72)***	−2.15 (4.04)***

* *P*<0.05,

** *P*<0.01,

*** *P*<0.001

Mean change from baseline (SD).

Infliximab significantly reduced the RAMRIS scores for both synovitis and osteitis in the wrist and MCPs as early as 2 weeks, and maintained reduction through 14 weeks (*P*<0.001, Table 2).

### Clinical outcomes

Infliximab reduced DAS28(CRP) disease activity at weeks 2, 4 and 14 compared with placebo (Table 2). As expected, placebo treatment also resulted in improvement in DAS28(CRP), but only approximately 40% as much as infliximab treatment.

### Association between baseline gene expression and 14-week response to infliximab

**Figure 1:**
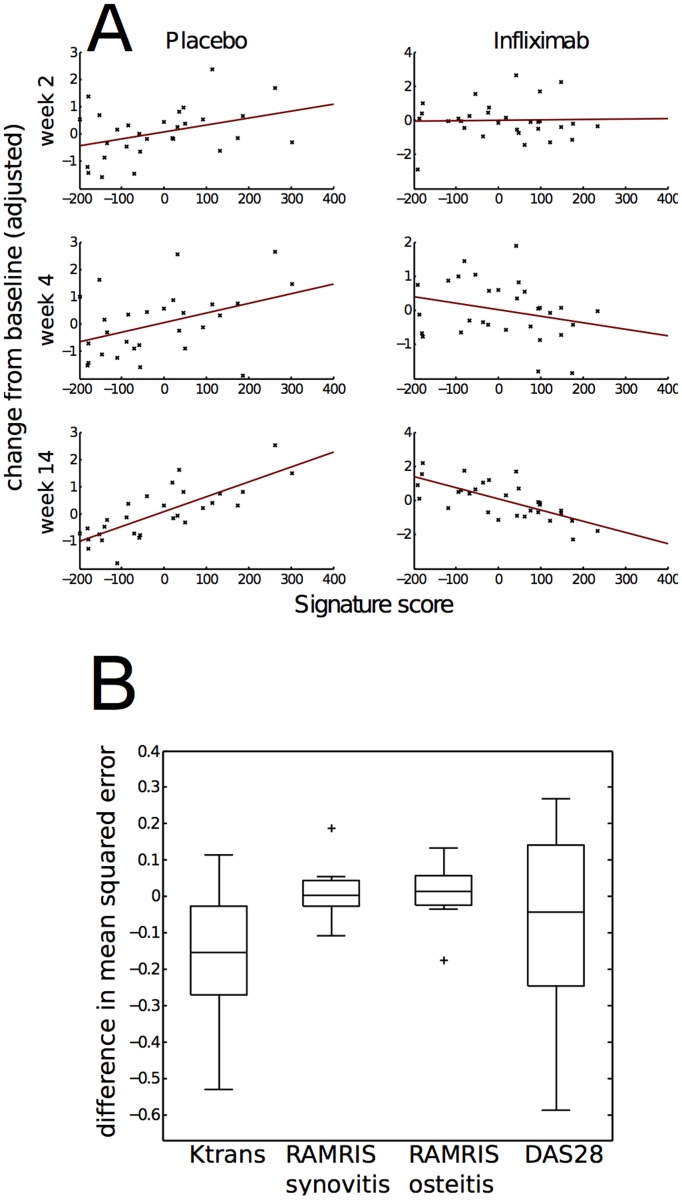
Gene expression is associated with change in disease activity measured by DCE-MRI. (A) High signature score correlates with K_trans_ improvement in the treatment arm and K_trans_ deterioration in the placebo arm. Scatter plots show baseline and treatment adjusted 14-week change in log Ktrans vs. signature score in both the treatment and placebo arms at weeks 2, 4, and 14. Linear models including terms for baseline K_trans_, treatment allocation, signature score, and the interaction between signature and treatment were fit to log K_trans_ change from baseline at each week. At both week 4 and 14, the signature score main effect and interaction with treatment were significant at p<0.05. (B) Whole blood gene expression improves prediction of week 14 change in K_trans_. In ten repeated rounds of random subsampling, 40 patients were selected and their whole blood gene expression data was used to identify genes associated with treatment response measured by K_trans_, DAS28(CRP), and RAMRIS. A linear model including terms for baseline disease activity, treatment allocation, signature score, and the interaction between signature and treatment was fit to week 14 data and used to predict week 14 changes for held out subjects. The distribution of mean squared prediction errors (MSE) minus the MSE achieved by a model excluding signature score terms is plotted for each endpoint. For K_trans_, but not DAS28(CRP), or RAMRIS, incorporation of baseline blood gene expression consistently improved prediction performance (p = 0.015, t-test).

We assessed the ability of baseline whole blood gene expression to predict 14-week change in K_trans_ by first applying linear regression to identify genes associated with response. The Q-Q plot of expected and observed p-values showed deviation from random expectation (Figure S1 in File S1). We then defined a novel gene signature consisting of 256 genes whose expression was statistically associated with 14-week change in K_trans_ of wrist (S1 Table). The signature score showed positive correlation (R^2^ = 0.51) with baseline-adjusted 14-week change in the infliximab-treatment arm and negative correlation (R^2^ = 0.62) in the placebo arm (Fig. 1A). Given the gradual reduction of K_trans_ of wrist with infliximab over time, we examined the association of the signature score with 4 week change in K_trans_, and found that the gene signature was statistically associated with 4-week change in K_trans_ data (p = 6.1e-3 signature main effect, p = 4.7e-3 signature by treatment interaction term). Analogous coefficient estimates from a model fit to the 2-week response data were not significant, although the signature score main effect approached significance (p = 0.059). Although the signature was derived using K_trans_ of wrist, the signature was also significantly associated with change in K_trans_ in the MCP joints at both 14 weeks (p = 0.013 main effect, p = 9.5e-5 interaction) and 4 weeks (p = 0.016 main effect, p = 9.5e-4 interaction). Next, by repeated random sampling of training and test sets, we estimated whether inclusion of baseline gene expression in a regression model improved prediction of 14-week change in K_trans_. For K_trans_, gene expression improved mean-squared prediction error of held out data (p<0.015, t-test) (Fig. 1B).

### Association between baseline gene expression and secondary endpoints

Similar analyses attempting to derive a gene signature using disease activity endpoints RAMRIS osteitis, RAMRIS synovitis, and DAS28(CRP) revealed no deviation from random expectation in Q-Q plots and no improvement in prediction of 14-week change in disease activity. There was no significant association between the K_trans_ signature score and change in DAS28(CRP), RAMRIS synovitis, or RAMRIS osteitis at any time point. However, at 14 weeks and only in the infliximab treatment arm, we identified a weak positive correlation between signature score and change in DAS28(CRP) (R^2^ = 0.12, P = 0.033 one-tailed) despite the poor correlation (R^2^ = 0.01, not significant) between 14-week change in DAS28 and change in log K_trans_. In the infliximab treatment arm we also examined the distribution of signature scores across EULAR response categories (an assessment of response based on pre-treatment DAS28 and post-treatment change in DAS28) [18]. Although the median signature score was higher in EULAR good and moderate responders than in non-responders, the difference was not significant by a Mann-Whitney U-test (P = 0.079).

### Association between predictive signature and response in previously published studies

**Figure 2:**
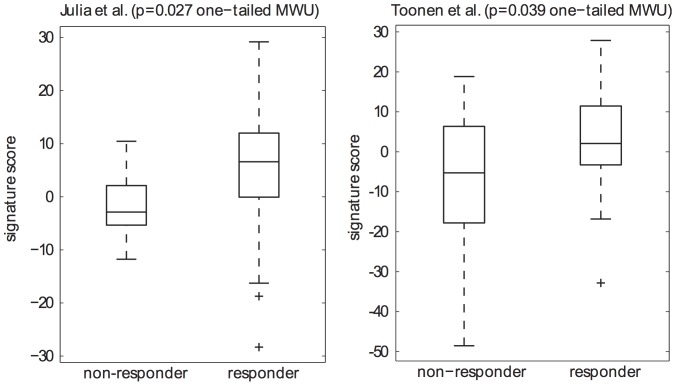
Gene signature score is higher in EULAR responders than non-responders in two independent studies. The predictive signature is associated with clinical response in two additional whole blood gene expression studies. The distribution of predictive signature scores was compared in responder and non-responder groups in the studies of Julia et al. and Toonen et al. In both cases, a one-tailed t-test identified statistically significant (p<0.05) differences.

We examined the predictive ability of our signature to categorize clinical response using previously reported data from studies where baseline whole blood gene expression was measured in the context of anti-TNF biologic treatment, and where gene expression data passed quality control [3], [10]. Signature scores were compared across patients stratified by EULAR response criteria. In both studies we observed a significantly higher signature score at baseline in responders compared to non-responders (Fig. 2). However, by calculating the area under a receiver operating characteristic curve (AUC) we determined that the ability of the signature to discriminate EULAR responders and non-responders was relatively poor. For the Julia study [3], the AUC was 0.73 (P = 0.056) and for the Toonen study [10] it was 0.66 (P = 0.080).

To examine whether infliximab treatment affects the expression level of genes in the predictive signature, we examined the effect of 1 month of infliximab treatment on expression of the 256 genes using the whole blood gene expression data set of Van Baarsen et al. [9]. Of the 13,027 detectably expressed genes in our study 10,059 were measured pre- and post-dose in whole blood by Van Baarsen and colleagues. Of these, 149 were significantly (p<0.01) up-regulated after infliximab treatment, whereas 1,370 were down-regulated. The median change in expression for all genes was a 1.1-fold down-regulation. Comparing the infliximab-induced fold changes observed for genes in our predictive signature with the overall distribution revealed significant differences (Figure S2 in File S1). Genes positively correlated with 14-week K_trans_ improvement showed greater median down-regulation (median 1.24-fold, p = 2.3e-8 by Mann-Whitney U-test) and 27/72 were significantly down-regulated by infliximab treatment (p = 3.2e-7 Fisher's exact test). Conversely, genes negatively correlated with 14-week K_trans_ improvement had higher median fold changes compared to all detected genes (median 1.05-fold down, p = 4.0e-3 by Mann-Whitney U-test), and were underrepresented in significantly down-regulated genes (9/122, p = 0.01 Fisher's exact test).

### Assessment of previously published predictive signatures

We assessed the ability of previously reported blood gene expression signatures to predict anti-TNF biologic response by evaluating their correlation with change in disease activity as measured by DCE-MRI, RAMRIS or DAS28(CRP). None of the previously reported gene signatures in Table 3 correlated with response endpoints in this study. Similarly, assessment of signature scores across patients grouped by EULAR response category showed no significant association between these pre-specified signatures and infliximab response (data not shown).

**Table 3:** Correlation coefficient (p-value) of baseline signature score and 14-week change in log K_trans_ of wrist and DAS28(CRP) for previously reported blood gene signatures.

Gene signature	DAS28(CRP) infliximab	DAS28(CRP) placebo	log K_trans_ infliximab	log K_trans_ placebo
Lequerré (8-gene)	0.18 (0.34)	−0.03 (0.88)	−0.05 (0.79)	−0.38 (0.04)
Lequerré (20-gene)	0.0 (1.0)	0.25 (0.19)	0.14 (0.46)	−0.27 (0.16)
Stuhlmuller	0.0 (1.0)	0.05 (0.80)	0.03 (0.87)	0.0 (1.0)
Sekiguchi	0.10 (0.60)	0.15 (0.44)	0.0 (1.0)	−0.27 (0.16)
Julia	0.0 (1.0)	−0.13 (0.50)	0.04 (0.83)	−0.07 (0.72)
Tanino	−0.04 (0.83)	−0.15 (0.44)	−0.13 (0.49)	0.0 (1.0)
Bienkowska	0.0 (1.0)	−0.12 (0.54)	−0.2 (0.30)	0.18 (0.35)

### Gene expression characteristics associated with infliximab response

A number of genes in the signature have been genetically associated with RA. Ten genes (ACAT1, AFF3, ANKRD55, CORO1C, FAM167A, FCGR3B, FCRL1, FCRLA, IKZF3, and TPD52) are located within RA genetic risk loci, and of these AFF3, ANKRD55, FCGR3B, FCRL1, FCRLA, and IKZF3 were reported to have additional supporting biological evidence linking them to the disease in a recent genetic and bioinformatic analysis [19]. Several other signature genes have been identified as biomarkers in RA or as being involved in the pathogenesis of the disease including vimentin [20], IL7R [21], CXCR4 [22], and platelet factor 4 [23]. To further explore the biology of the predictive signature, we used unsupervised clustering to group the 256 genes into four clusters with highly correlated expression across the 59 patient samples (Figure S3 in File S1). We annotated the clusters by examining their statistical enrichment for relevant blood gene expression signatures, canonical pathways and biological processes, and by assessing their correlation with pre-specified blood gene expression modules described by Chaussabel and coworkers [24]. The clusters and annotations are summarized in Table 4. Assignment of genes to clusters is provided in S1 Table.

**Table 4:** Gene clusters comprising the predictive signature.

Cluster	Description	Expression in responders	Representative gene sets (enrichment p-value)	Most correlated blood module (Chaussabel et al.)
1	Platelets	Higher	Platelet aggregation (6.2e-9)	M 1.2 Platelets (R^2^ = 0.87)
2	Myeloid cells	Higher	Up-regulated in whole blood at day 3 of septic shock in children (6.3e-15) Up-regulated in unpurified PBMC vs. naïve T-cell fraction (2.5e-18)	M 1.5 Myeloid/monocytes (R^2^ = 0.61)
3	B-cells	Lower	B-cell differentiation (5.1e-7)	M 1.3 B-cells (R^2^ = 0.81)
4	G-CSF down-regulated	Lower	Down-regulated in peripheral blood after administration of G-CSF (1.8e-27) Nucleic acid metabolism (3.5e-5)	M 1.8 Metabolism (R^2^ = 0.65)

Expression of genes in two clusters correlated positively with infliximab response. Cluster 1 is highly concordant with the Chaussabel platelet module and is enriched for platelet markers and genes involved in platelet aggregation like calmodulin-3, CD36, platelet factor 4, prostacyclin receptor, and thromboxane A2 receptor. Cluster 2 is concordant with the Chaussabel myeloid/monocyte blood module and contains genes expressed specifically in myeloid lineage cells including the low affinity Fcγ receptor IIIb, CD14, CD300LB, and NQO2.

In contrast, clusters 3 and 4 are comprised of genes whose expression levels correlate with infliximab non-response. Cluster 3 is enriched for markers of activated B-cells and plasma cells including B-cell linker, IGLJ3, CD79b, CD72, early B-cell factor 1, and IKZF3 and consequently shows high correlation with the B-cell blood module. Cluster 4 is enriched for genes down-regulated in human blood after administration of recombinant G-CSF, a cytokine that mobilizes myeloid cells from the bone marrow. This cluster shows the highest correlation with a metabolism-related Chaussabel blood module.

## Discussion

Clinical characteristics explain only a small portion of the total variability in patient response to anti-TNF treatment in RA [1], [25]. Previous studies have explored the possibility that inadequate responders comprise a molecular subtype that can be identified using blood mRNA profiling but no gene expression biomarkers have been convincingly validated. This may be attributable to limitations in both study design and the endpoints used to evaluate clinical response. Most placebo controlled clinical studies of RA disease activity, including this one, report a substantial placebo effect. Additionally, RA patients have a fluctuating disease course, and enrollment of patients at the apogee of disease activity may account for up to a third of the total DAS28 improvement in response to TNFα inhibitors [26]. However, no prior anti-TNF biomarker study has examined blood mRNA from placebo-treated patients, potentially confounding treatment-dependent and independent disease improvement. Indeed, the single signature reported to be validated by Toonen et al. [10] [19]showed the highest correlation with baseline DAS28 of any signature examined in this study (R^2^ = 0.137, P = 0.004) suggesting that its reported correlation with response may be explained, in part, by enriching for patients with flaring disease. More objective measures of RA disease activity and progression could aid in the identification of predictive biomarkers [27], [28], and objective measures of joint inflammation are expected to associate more robustly with molecular predictors of response to a TNF blocker like infliximab.

In this study, we propose a path forward by profiling gene expression in both placebo and treatment arms and by employing a sensitive, quantitative, and objective DCE-MRI endpoint: K_trans_ of synovium in the wrist. In contrast to the standard clinical endpoint DAS28, DCE-MRI showed no improvement with placebo. Using DCE-MRI we derived a pre-treatment whole blood gene expression signature correlating with change in the imaging biomarker K_trans_. The signature was associated with K_trans_ improvement in infliximab-treated patients but with worsening K_trans_ in the placebo arm. A similar association was seen with K_trans_ summed from the metacarpophalangeal joints of the hand in each treatment arm.

We observed no correlation between the gene expression signature and 14-week change in RAMRIS synovitis or osteitis. This observation is potentially unexpected, because RAMRIS synovitis scores and K_trans_ both represent gadolinium contrast transfer to synovium, and both have been validated as inflammation biomarkers through clinical association with joint swelling and with inflammatory pathology on biopsy [29]. However, histological inflammation of cellular infiltration has in several studies been more strongly associated with dynamic measures of gadolinium transfer (e.g. K_trans_ and the rate of early enhancement) as opposed to static images obtained at the peak of contrast enhancement used for RAMRIS methodology [12], [30]. Further, several studies have suggested that DCE-MRI is a more sensitive biomarker of joint inflammation than RAMRIS, demonstrating better correlation with clinical and laboratory markers of active disease and response to treatment [31], [32]. Although RAMRIS and DCE-MRI correlate and are valid measures for detecting treatment change, they are not interchangeable and the more direct link between dynamic MRI and the pathophysiology of vascularity and joint inflammation may make it more suitable for the identification of blood gene expression markers of treatment response.

Interestingly, evaluation of the effect of 1 month of infliximab treatment on the genes in the signature revealed evidence of infliximab-induced gene expression changes for many genes. In particular, genes whose expression correlated with K_trans_ improvement after infliximab treatment were highly enriched for genes reported to be down-regulated by treatment. This observation provides confidence that the genes in our predictive signature are related to the biology of anti-TNF treatment in rheumatoid arthritis.

Unfortunately, prior gene expression studies have not employed DCI-MRI as an endpoint. DCE-MRI is infrequently used in studies of RA patients, and so independent validation of our signature and K_trans_ response in other patients will need to await the availability of other datasets. However, in both studies we examined EULAR responders had significantly higher signature scores than non-responders, consistent with expectation. Although the higher signature score observed in EULAR responders is interesting, the poor discrimination achieved using the signature to distinguish responders and non-responders indicates it would be of limited use in clinical practice. Nevertheless, to the extent that the signature genuinely defines molecular subtypes in RA it could serve as an interesting endpoint in future biomarker or genetic studies of the disease. One potentially interesting avenue would be to examine the association of genetic variants with gene signature score to identify genetic drivers of the putative patient subtypes reported here. In addition, by combining all genes into a single score representing an average log expression level, we have ignored the possibility that individual genes identified in this study may have greater predictive value than others. It would be interesting to explore predictive modeling approaches that take this into account.

Unsupervised clustering of the genes making up the predictive signature described here suggests that they broadly reflect three sources: 1) myeloid lineage cells 2) B-cells, and 3) reticulated platelets. High expression of myeloid cell markers and low expression of genes down-regulated by recombinant G-CSF in peripheral blood is associated with response to infliximab in RA. Although *in vitro* TNF inhibits hematopoiesis, chronic inflammation in RA can be associated with monocytosis and granulocytosis [33], [34], and TNF induces production of pro-myelopoietic factors like G-CSF [35] and GM-CSF [36]. High expression of platelet and platelet activation markers also correlates with good infliximab response. Whole blood mRNA profiling can detect transcripts from reticulated platelets whose level in blood is indicative of the level of thrombopoiesis and platelet turnover. Platelets have a well-characterized role in RA pathogenesis by promoting vascular permeability in the inflamed joint, releasing prostaglandins, and shedding IL1-containing vesicles that promote inflammation [37]. TNF impacts platelet function by enhancing their activation [38] and promoting megakaryocytopoiesis [39]. Further, TNF has been demonstrated to shorten platelet life [40]–[42]. Finally, high expression of B-cell and plasma cell markers correlates with poor response to infliximab. Interestingly, TNF is reported to mediate loss of B-cell lymphopoiesis [43]. It is also notable that one report has suggested that RA patients who fail on a TNF blocker show better response to the B-cell depletion therapy Rituximab than to a second anti-TNF [44].

Taken together, these results are consistent with the hypothesis that infliximab responders have higher levels of inflammatory processes driven by TNF than non-responders. In this model, anti-TNF responders may show increased induction of pro-myelopoietic factors which mobilize granulocytes and monocytes from the bone marrow, and collaborate with TNF to inhibit B-cell lymphopoiesis and increase megakaryocytopoiesis, platelet turnover, and hence the fraction of reticulated platelets in whole blood. These effects would be reflected in the myeloid, B-cell, and platelet components of the baseline whole blood gene expression signature we report here.

## Conclusions

This study provides evidence that a whole blood gene expression signature correlates with response to infliximab as assessed by DCE-MRI. High signature score associated with improvement in infliximab-treated patients but not in placebo-treated patients, however the ability of the signature to stratify EULAR responders and non-responders was poor. Additional studies should attempt to validate this finding and explore the molecular underpinnings of the gene signature and its relationship to anti-TNF response, in particular the hypothesis that anti-TNF inadequate responders comprise a distinct molecular subtype in RA.

## Supporting Information

S1 File **Supplemental figures and methods.** Figure S1, Q-Q plots for primary and secondary response endpoints. Linear regression was applied to identify genes associated with treatment-specific response to infliximab. Observed p-values for the gene expression by treatment interaction coefficients are plotted vs. random expectation based on a uniform null distribution for each response endpoint. Figure S2, Histograms of gene expression log fold changes observed after one month of infliximab treatment in the study of Van Baarsen et al. From top to bottom: all genes with detectable expression in whole blood, predictive signature genes whose expression is correlated with 14-week log K_trans_ improvement in infliximab-treated patients, and signature genes whose expression is anti-correlated with 14-week change in log K_trans_. Figure S3, Heat map of clustered signature genes. Predictive signature genes were clustered into four groups based on their correlation in expression. Heat map color indicates the correlation in expression between genes across all patients' baseline whole blood samples.
(DOCX)

S1 Table **Genes associated with 14-week change in RA disease activity measured by DCE-MRI.**
(DOCX)
