# Supplementary material for: Pre-Treatment Whole Blood Gene Expression Is Associated with 14-Week Response Assessed by Dynamic Contrast Enhanced Magnetic Resonance Imaging in Infliximab-Treated Rheumatoid Arthritis Patients
Source: PLoS One. 2014 Dec 12;9(12):e113937. doi: 10.1371/journal.pone.0113937 (PMC4264695; doi:10.1371/journal.pone.0113937)
Supplement: S1 File — Supplemental figures and methods. Figure S1, Q-Q plots for primary and secondary response endpoints. Linear regression was applied to identify genes associated with treatment-specific response to infliximab. Observed p-values for the gene expression by treatment interaction coefficients are plotted vs. random expectation based on a uniform null distribution for each response endpoint. Figure S2, Histograms of gene expression log fold changes observed after one month of infliximab treatment in the study of Van Baarsen et al. From top to bottom: all genes with detectable expression in whole blood, predictive signature genes whose expression is correlated with 14-week log Ktrans improvement in infliximab-treated patients, and signature genes whose expression is anti-correlated with 14-week change in log Ktrans. Figure S3, Heat map of clustered signature genes. Predictive signature genes were clustered into four groups based on their correlation in expression. Heat map color indicates the correlation in expression between genes across all patients' baseline whole blood samples. (DOCX) [file pone.0113937.s001.docx]

**Figure S1: Observed p-values for gene expression*treatment interaction coefficients vs. random expectation based on a uniform null distribution for each response endpoint.**

**Figure S2 – Histogram of fold changes observed after one month of infliximab treatment in the study of Van Baarsen et al. From top to bottom: all genes with detectable expression in whole blood, predictive signature genes whose expression is correlated with 14-week K_trans_ improvement in infliximab-treated patients, and signature genes whose expression is anti-correlated with 14-week change in K_trans_.**

**Figure S3 – Heat map of clustered signature genes. Heat map color indicates the correlation in expression between genes across all patients’ baseline whole blood samples.**


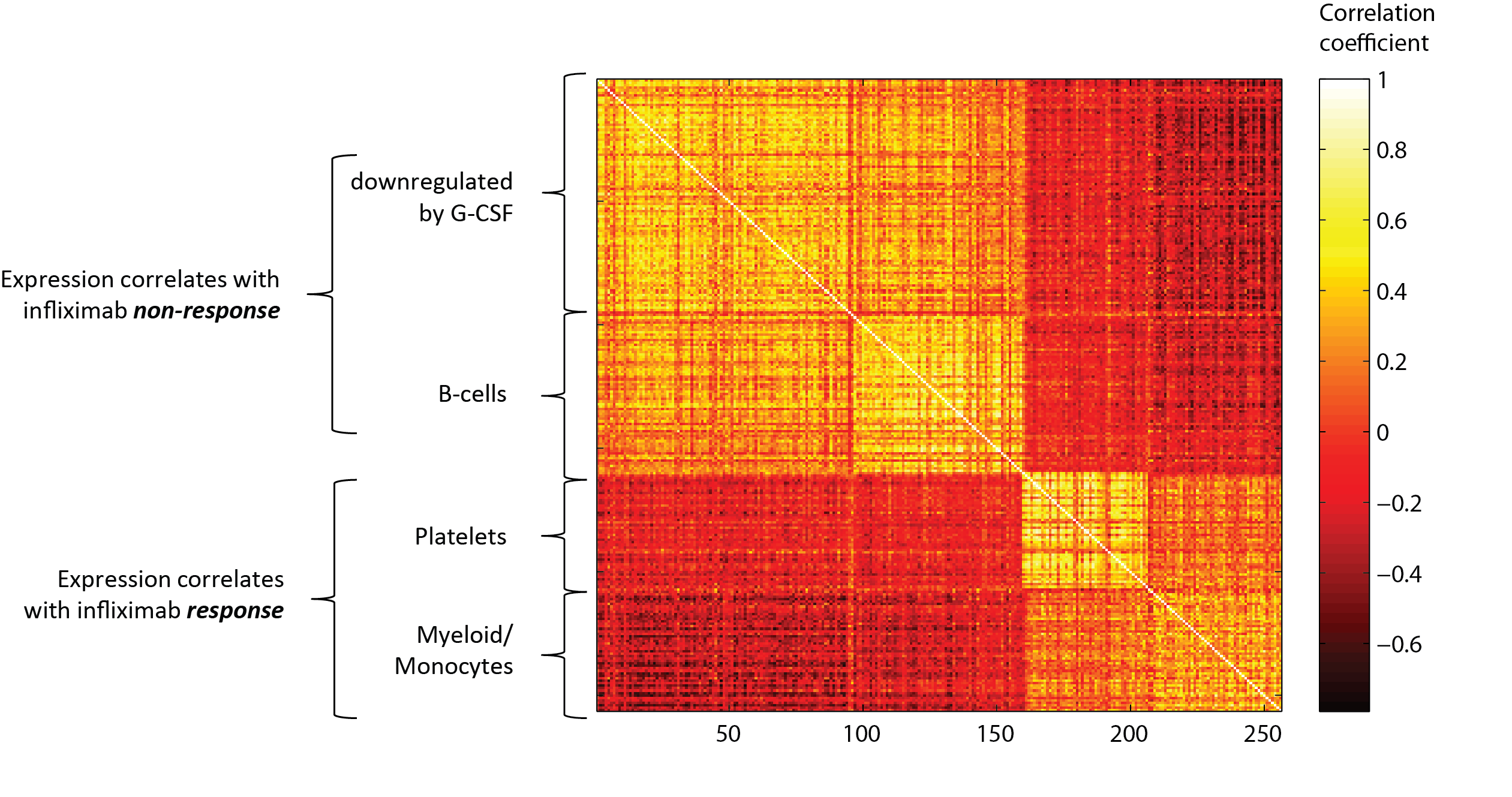


**Supplemental Methods**

***Subjects***

Male and female participants at least 18 years of age, with a diagnosis of RA for at least 6 months (based on the American College of Rheumatology (ACR) 1987 criteria), at least 6 tender and 6 swollen joints (using the 28 joint set), C-reactive protein (CRP) ≥1.0 mg/L or an Erythrocyte Sedimentation Rate ≥28 mm/hour, and who were on a stable dose of methotrexate, were recruited at four clinical sites in Europe. All subjects were naïve to anti-TNF biologics, and were required to have RAMRIS synovitis score ≥ 1 in the radio-carpal or intercarpal joints of one hand based on centralized expert assessment. Other requirements for enrollment included adequate hematological status, aspartate and alanine aminotransferase levels ≤2.5 times the upper limit of normal, and a clinically acceptable electrocardiogram. In order to minimize risks associated with GBCA, subjects with evidence of renal insufficiency or history of adverse reaction to GBCA were excluded. Patients had no contraindications to infliximab or MRI. All subjects provided written informed consent. Sample size was selected to give 80% power to detect treatment effect based on an estimated treatment effect size of 0.7 to 0.8 on DAS28.

***Treatment***

At weeks 0, 2, 6, and 14, participants received 250 mL of either infliximab 3 mg/kg in 0.9% NaCl or 0.9% NaCl alone, infused over a 2-hour period. Subjects Patients continued to receive their standard dose and regimen of disease modifying antirheumatic drugs (DMARDS) (e.g. methotrexate and folate), nonsteroidal anti-inflammatory drugs (NSAID) or cyclo-oxygenase inhibitors (COXibs), and/or glucocorticoid (e.g. prednisone as long as the dose was ≤ 10 mg per day). After 14 weeks of double-blinded therapy, participants in consultation with their physician could elect 3 months of open label infliximab treatment following the labeled dosing recommendations based on their previous randomized treatment assignment.

***Clinical assessments***

DAS28(CRP) is a composite score of the number of tender joints (28 joint count), the number of swollen joints (28 joint count), patient global assessment of disease (GADP) on a 100 mm visual analog scale (VAS), and CRP (mg/dL). Joint counts were performed by examiners masked to treatment assignment who did not serve as study physicians.

***MRI assessments***

MRI of the most clinically involved hand and wrist was acquired at baseline and weeks 2, 4, and 14 in order to measure Ktrans. The wrist, MCP and proximal interphalangeal (PIP) joints were included within a single field of view (FOV) using a commercial multi-channel knee coil [1]. An acrylic frame [2] was used to ensure fixed, reproducible positioning of the hand and wrist joints on serial MRI examinations. Two small tubes containing solutions of copper sulfate sufficient to provide T1 values at 1.5T of 90ms and 1080ms were placed alongside the index and fifth fingers to serve as T1 standards for DCE-MRI measurements. The MRI protocol began with coronal short-tau inversion recovery (STIR) (repetition time [TR] of 3,000 ms, echo time [TE] of 38 ms, inversion time [TI] of 150 ms, 20 contiguous 3-mm slices, FOV of 18 cm x 18 cm, matrix of 384 x 384) and coronal three-dimensional (3D) T1-weighted gradient-echo (GRE) (TR of 29 ms, TE of 12 ms, flip angle of 20 degrees, 40 slices of 1.5-mm thickness, FOV of 18 cm x 18 cm, matrix of 512 x 256, selective water excitation). These were followed by a DCE-MRI sequence composed of 35 sequential 3D T1-weighted GRE (TR of 4.2 ms, TE of 1.7 ms, flip angle of 30 degrees, 30 slices of 2.0-mm thickness, FOV of 18 cm x 13.5 cm, matrix of 192 x 144, selective water excitation; 9 sec/scan), with infusion of gadolinium diethylenetriaminepentacetate (Gd-DTPA) (Magnevist, Germany) (0.1 mg/kg, 0.2 cc/kg injected at 3 cc/sec, followed by a 20 cc saline flush) using a power injector after the sixth sequential scan. The DCE-MRI sequence was followed by coronal 3D T1-weighted GRE (TR of 29 ms, TE of 12 ms, flip angle of 20 degrees, 40 slices of 1.5-mm thickness, FOV of 18 cm x 18 cm, matrix of 512 x 256, selective water excitation) and axial 3D T1-weighted GRE (TR of 29 ms, TE of 12 ms, flip angle of 20 degrees, 90 slices of 2.0-mm thickness, FOV of 18 cm x 9 cm and matrix of 384 x 154, selective water excitation).

Small ROIs were placed manually over areas of enhancing synovium or enhancing tissue (synovitis and osteitis) without knowledge of the examination dates or the order in which examinations were acquired. Care was taken to ensure that identical locations were selected for each study visit of an individual subject. The rate of GBCA synovial leakage was measured using a pharmacokinetic compartment model [3] that quantifies the exchange of contrast agent between the plasma and tissue extracellular space (synovium). The model requires a measure of the rate of GBCA input into the plasma which is controlled with a power injector and derived using an automated method [4] from T1 signal intensity in the radial artery over time. Outputs of the model include Ktrans (sec-1), a rate constant that reflects the flow and permeability surface area of enhancing synovium (primary endpoint) and enhancing tissue (synovitis and osteitis).

RAMRIS [5] of synovitis, osteitis and bone erosion was determined at baseline, 2, 4 and 14 weeks by two independent radiologists blinded to visit order and treatment assignments.

***Gene signature clustering***

The Pearson correlation matrix for all 256 genes in the predictive signature was calculated. Gene-gene similarity for genes i and j, si,j, was defined as mutual information weighted by the sign of the correlation ri,j, assuming a joint normal distribution in their expression across all patients:

$$s_{i,j}=-\frac{\mathrm{sign}\left( r_{i,j} \right)}{2}\log\left( 1-r_{i,j}^{2} \right)$$

The resulting similarity matrix was input to the affinity propagation (AP) algorithm to cluster the genes [6]. The AP self-similarity parameter was adjusted to obtain a variety of deterministic clustering solutions varying from a single cluster to 256 single-gene clusters. The preferred number of clusters was selected using a minimum description length heuristic. Each clustering was used to obtain parameter estimates for a mixture of Gaussians distribution using the EM algorithm, with the number of mixture components equal to the number of clusters. The description length (i.e. log posterior probability) of the observed data was calculated for each mixture distribution. The solution with four clusters minimized the description length.

Reference List

1. DiCarlo JC, Hargreaves BA, Butts Pauly K, J Baslio, PJPCG Countryman. MRI technique for rheumatoid arthritis clinical trials shortens hand/wrist imaging time. Presented at European League Against Rheumatism; Annual European Congress of Rheumatology (EULAR), Berlin Germany. 2012.

2. CG Peterfy, E Olech, JC DiCarlo, JT Merrill, PJ Countryman, NB Gaylis: **Monitoring cartilage loss in the hands and wrists in rheumatoid arthritis with magnetic resonance imaging in a multi-center clinical trial: IMPRESS (NCT00425932).** *Arthritis Res Ther* 2013, **15:** R44.

3. PS Tofts, G Brix, DL Buckley, JL Evelhoch, E Henderson, MV Knopp, HB Larsson, TY Lee, NA Mayr, GJ Parker et al.: **Estimating kinetic parameters from dynamic contrast-enhanced T(1)-weighted MRI of a diffusable tracer: standardized quantities and symbols.** *J Magn Reson Imaging* 1999, **10:** 223-232.

4. E Ashton, D Raunig, C Ng, F Kelcz, T McShane, J Evelhoch: **Scan-rescan variability in perfusion assessment of tumors in MRI using both model and data-derived arterial input functions.** *J Magn Reson Imaging* 2008, **28:** 791-796.

5. M Ostergaard, C Peterfy, P Conaghan, F McQueen, P Bird, B Ejbjerg, R Shnier, P O'Connor, M Klarlund, P Emery et al.: **OMERACT Rheumatoid Arthritis Magnetic Resonance Imaging Studies. Core set of MRI acquisitions, joint pathology definitions, and the OMERACT RA-MRI scoring system.** *J Rheumatol* 2003, **30:** 1385-1386.

6. BJ Frey, D Dueck: **Clustering by passing messages between data points.** *Science* 2007, **315:** 972-976.
