# Supplementary material for: Pre-Treatment Whole Blood Gene Expression Is Associated with 14-Week Response Assessed by Dynamic Contrast Enhanced Magnetic Resonance Imaging in Infliximab-Treated Rheumatoid Arthritis Patients
Source: PLoS One. 2014 Dec 12;9(12):e113937. doi: 10.1371/journal.pone.0113937 (PMC4264695; doi:10.1371/journal.pone.0113937)
Supplement: S1 Table — Genes associated with 14-week change in RA disease activity measured by DCE-MRI. (DOCX) [file pone.0113937.s002.docx]

**Table S1 – Predictive gene signature**

| *Gene* | *Cluster* | *Expression*Treatment interaction coefficient estimate* | *Standard error* | *p-value* |
| --- | --- | --- | --- | --- |
| ABCC3 | Platelets | 0.65 | 0.20 | 1.63E-03 |
| ABCD2 | GCSF down | -0.56 | 0.21 | 9.79E-03 |
| ACAT1 | GCSF down | -0.58 | 0.21 | 6.56E-03 |
| AFF3 | B-cells | -0.57 | 0.20 | 6.54E-03 |
| AIFM2 | B-cells | -0.55 | 0.20 | 7.02E-03 |
| ALG5 | GCSF down | -0.69 | 0.20 | 1.17E-03 |
| ANKRD55 | Platelets | 0.58 | 0.19 | 4.44E-03 |
| ANXA5 | Myeloid | 0.61 | 0.21 | 5.97E-03 |
| AQP10 | Platelets | 0.59 | 0.20 | 4.80E-03 |
| ARHGAP6 | Platelets | 0.68 | 0.19 | 7.16E-04 |
| ARRB2 | Myeloid | 0.66 | 0.19 | 1.23E-03 |
| ASAP2 | Platelets | 0.53 | 0.19 | 7.30E-03 |
| ASF1A | GCSF down | -0.62 | 0.21 | 5.13E-03 |
| ATR | GCSF down | -0.63 | 0.20 | 2.10E-03 |
| BBS4 | B-cells | -0.62 | 0.21 | 4.93E-03 |
| BCL7A | B-cells | -0.63 | 0.20 | 2.72E-03 |
| BEND5 | B-cells | -0.73 | 0.19 | 3.24E-04 |
| BLNK | B-cells | -0.60 | 0.20 | 4.63E-03 |
| BTLA | B-cells | -0.64 | 0.23 | 6.59E-03 |
| C10orf11 | Myeloid | 0.53 | 0.20 | 9.84E-03 |
| C12orf11 | GCSF down | -0.64 | 0.20 | 1.84E-03 |
| C20orf117 | Myeloid | 0.69 | 0.20 | 1.02E-03 |
| C7orf13 | GCSF down | -0.75 | 0.19 | 2.14E-04 |
| C8orf46 | Platelets | 0.64 | 0.20 | 1.69E-03 |
| CALM3 | Platelets | 0.66 | 0.20 | 1.54E-03 |
| CAPN1 | Myeloid | 0.64 | 0.21 | 3.46E-03 |
| CBFA2T2 | B-cells | -0.58 | 0.20 | 5.47E-03 |
| CBLB | GCSF down | -0.62 | 0.20 | 2.88E-03 |
| CCDC152 | GCSF down | -0.60 | 0.21 | 5.12E-03 |
| CCDC76 | GCSF down | -0.61 | 0.22 | 7.74E-03 |
| CCT2 | GCSF down | -0.59 | 0.20 | 5.28E-03 |
| CD14 | Myeloid | 0.59 | 0.21 | 6.25E-03 |
| CD151 | Platelets | 0.68 | 0.22 | 3.70E-03 |
| CD300LB | Myeloid | 0.81 | 0.21 | 2.67E-04 |
| CD36 | Platelets | 0.60 | 0.21 | 6.79E-03 |
| CD52 | GCSF down | -0.63 | 0.21 | 4.24E-03 |
| CD72 | B-cells | -0.56 | 0.20 | 7.53E-03 |
| CD79B | B-cells | -0.56 | 0.20 | 7.54E-03 |
| CHRAC1 | Platelets | 0.64 | 0.21 | 3.15E-03 |
| CHST2 | Myeloid | 0.61 | 0.20 | 2.82E-03 |
| CLDN5 | Platelets | 0.57 | 0.20 | 6.70E-03 |
| CLDND1 | GCSF down | -0.57 | 0.21 | 9.60E-03 |
| CLLU1 | B-cells | -0.63 | 0.20 | 2.63E-03 |
| CMTM7 | B-cells | -0.56 | 0.20 | 7.40E-03 |
| CORO1C | Myeloid | 0.60 | 0.20 | 4.07E-03 |
| COTL1 | Myeloid | 0.61 | 0.22 | 6.76E-03 |
| CRHR1 | Myeloid | 0.64 | 0.20 | 2.23E-03 |
| CTSZ | Myeloid | 0.56 | 0.21 | 9.46E-03 |
| CXCR2P1 | Platelets | 0.59 | 0.21 | 6.03E-03 |
| CXCR4 | GCSF down | -0.60 | 0.20 | 5.10E-03 |
| CXCR6 | GCSF down | -0.61 | 0.20 | 3.11E-03 |
| DBF4 | GCSF down | -0.57 | 0.20 | 7.17E-03 |
| DBNL | Myeloid | 0.62 | 0.21 | 4.44E-03 |
| DHRS7B | Myeloid | 0.55 | 0.20 | 7.53E-03 |
| DNAJC8 | GCSF down | -0.56 | 0.20 | 7.03E-03 |
| DOPEY1 | GCSF down | -0.65 | 0.21 | 3.06E-03 |
| EAF2 | GCSF down | -0.60 | 0.22 | 7.49E-03 |
| EBF1 | B-cells | -0.54 | 0.20 | 9.23E-03 |
| EBPL | GCSF down | -0.61 | 0.20 | 4.49E-03 |
| EIF2A | B-cells | -0.61 | 0.23 | 9.60E-03 |
| ELOVL7 | Platelets | 0.57 | 0.20 | 6.79E-03 |
| ELP2 | GCSF down | -0.56 | 0.21 | 8.04E-03 |
| EMILIN2 | Myeloid | 0.65 | 0.20 | 1.84E-03 |
| ENAM | B-cells | -0.56 | 0.20 | 7.10E-03 |
| ESAM | Platelets | 0.57 | 0.20 | 5.50E-03 |
| F13A1 | Platelets | 0.58 | 0.20 | 4.43E-03 |
| FAM103A1 | Myeloid | 0.55 | 0.20 | 9.29E-03 |
| FAM127C | Myeloid | 0.62 | 0.20 | 3.20E-03 |
| FAM129C | B-cells | -0.61 | 0.20 | 3.61E-03 |
| FAM167A | B-cells | -0.54 | 0.20 | 9.55E-03 |
| FAM98A | GCSF down | -0.63 | 0.20 | 3.16E-03 |
| FASTKD1 | GCSF down | -0.58 | 0.20 | 5.88E-03 |
| FCGR3B | Myeloid | 0.57 | 0.20 | 6.41E-03 |
| FCRL1 | B-cells | -0.58 | 0.20 | 5.63E-03 |
| FCRLA | B-cells | -0.59 | 0.20 | 3.92E-03 |
| FLJ41649 | Myeloid | 0.64 | 0.20 | 2.13E-03 |
| FLJ44635 | B-cells | -0.55 | 0.20 | 9.58E-03 |
| FLJ45508 | Myeloid | 0.56 | 0.20 | 8.02E-03 |
| FTH1 | Myeloid | 0.71 | 0.21 | 1.65E-03 |
| GABARAP | Myeloid | 0.64 | 0.21 | 4.11E-03 |
| GAN | B-cells | -0.63 | 0.20 | 3.08E-03 |
| GAPDH | Myeloid | 0.55 | 0.20 | 7.92E-03 |
| GAS2L1 | Platelets | 0.63 | 0.20 | 2.26E-03 |
| GOLGA4 | GCSF down | -0.62 | 0.19 | 2.37E-03 |
| GOLPH3L | GCSF down | -0.71 | 0.21 | 1.20E-03 |
| GOT1 | B-cells | -0.61 | 0.21 | 4.66E-03 |
| GPR111 | Myeloid | 0.58 | 0.22 | 9.25E-03 |
| GTPBP8 | GCSF down | -0.53 | 0.20 | 9.71E-03 |
| GZMK | GCSF down | -0.57 | 0.21 | 8.81E-03 |
| HIBADH | GCSF down | -0.62 | 0.21 | 5.02E-03 |
| HIST1H2BD | Platelets | 0.68 | 0.21 | 1.88E-03 |
| HIST1H2BJ | Platelets | 0.59 | 0.21 | 7.02E-03 |
| HSPA1A | Myeloid | 0.62 | 0.22 | 8.03E-03 |
| HSPA8 | GCSF down | -0.61 | 0.20 | 2.94E-03 |
| IFT57 | B-cells | -0.70 | 0.22 | 2.08E-03 |
| IGLJ3 | B-cells | -0.57 | 0.21 | 9.00E-03 |
| IGLV3-25 | B-cells | -0.55 | 0.20 | 8.54E-03 |
| IKZF3 | B-cells | -0.66 | 0.19 | 1.18E-03 |
| IL28RA | B-cells | -0.68 | 0.22 | 2.92E-03 |
| IL7R | GCSF down | -0.57 | 0.20 | 5.99E-03 |
| ITM2C | B-cells | -0.58 | 0.21 | 7.35E-03 |
| KATNA1 | GCSF down | -0.64 | 0.20 | 2.79E-03 |
| KCND3 | Platelets | 0.63 | 0.21 | 3.59E-03 |
| KDELR1 | Myeloid | 0.55 | 0.20 | 9.04E-03 |
| KIAA0125 | B-cells | -0.62 | 0.20 | 3.69E-03 |
| LEFTY1 | Platelets | 0.64 | 0.20 | 2.08E-03 |
| LETMD1 | GCSF down | -0.56 | 0.20 | 6.78E-03 |
| LHX4 | B-cells | -0.57 | 0.21 | 9.72E-03 |
| LIMS1 | Platelets | 0.67 | 0.19 | 8.52E-04 |
| LIX1 | B-cells | -0.55 | 0.20 | 9.37E-03 |
| LMBR1 | GCSF down | -0.56 | 0.21 | 9.32E-03 |
| LOC100128252 | B-cells | -0.71 | 0.19 | 4.10E-04 |
| LOC100129858 | GCSF down | -0.68 | 0.19 | 7.70E-04 |
| LOC100271722 | Myeloid | 0.58 | 0.20 | 5.55E-03 |
| LOC100507387 | Myeloid | 0.68 | 0.21 | 1.96E-03 |
| LOC100527964 | GCSF down | -0.62 | 0.20 | 2.65E-03 |
| LOC152217 | GCSF down | -0.62 | 0.22 | 7.21E-03 |
| LOC157562 | B-cells | -0.65 | 0.20 | 1.59E-03 |
| LOC283663 | B-cells | -0.59 | 0.20 | 4.76E-03 |
| LOC285178 | B-cells | -0.58 | 0.20 | 5.24E-03 |
| LOC339192 | Myeloid | 0.54 | 0.20 | 8.57E-03 |
| LOC386758 | B-cells | -0.69 | 0.20 | 8.49E-04 |
| LOC644936 | Myeloid | 0.56 | 0.21 | 8.97E-03 |
| LOC729451 | Platelets | 0.68 | 0.20 | 1.11E-03 |
| MAFG | Myeloid | 0.57 | 0.21 | 7.39E-03 |
| MANF | GCSF down | -0.71 | 0.23 | 3.18E-03 |
| MAPK9 | GCSF down | -0.67 | 0.19 | 1.02E-03 |
| MARS2 | GCSF down | -0.57 | 0.20 | 6.89E-03 |
| MEFV | Myeloid | 0.64 | 0.21 | 4.05E-03 |
| METTL5 | GCSF down | -0.65 | 0.21 | 2.96E-03 |
| MFAP3L | Platelets | 0.71 | 0.19 | 5.17E-04 |
| MIOS | GCSF down | -0.57 | 0.20 | 5.95E-03 |
| MKI67IP | GCSF down | -0.58 | 0.21 | 7.00E-03 |
| MON1B | Myeloid | -0.55 | 0.20 | 7.80E-03 |
| MPL | Platelets | 0.61 | 0.21 | 5.76E-03 |
| MRPL18 | GCSF down | -0.56 | 0.20 | 6.85E-03 |
| MSTO1 | B-cells | -0.62 | 0.20 | 3.70E-03 |
| MYLK4 | GCSF down | 0.55 | 0.20 | 9.48E-03 |
| MYO1B | B-cells | -0.75 | 0.19 | 2.58E-04 |
| NAGK | Myeloid | 0.62 | 0.20 | 2.84E-03 |
| NCAPH | GCSF down | -0.56 | 0.20 | 8.67E-03 |
| NDFIP2 | GCSF down | -0.62 | 0.20 | 2.64E-03 |
| NECAB2 | Myeloid | 0.59 | 0.20 | 3.79E-03 |
| NKIRAS1 | GCSF down | -0.63 | 0.20 | 2.45E-03 |
| NOC3L | GCSF down | -0.59 | 0.22 | 8.07E-03 |
| NQO2 | Myeloid | 0.64 | 0.21 | 3.87E-03 |
| NT5E | B-cells | -0.64 | 0.19 | 1.78E-03 |
| NTNG1 | Myeloid | 0.74 | 0.19 | 2.51E-04 |
| NUF2 | GCSF down | -0.62 | 0.21 | 5.43E-03 |
| OFD1 | GCSF down | -0.61 | 0.21 | 4.95E-03 |
| ORC2 | GCSF down | -0.64 | 0.20 | 2.40E-03 |
| ORC3 | B-cells | -0.74 | 0.20 | 4.39E-04 |
| OSTF1 | Myeloid | 0.58 | 0.21 | 8.09E-03 |
| P2RX5 | B-cells | -0.62 | 0.20 | 2.68E-03 |
| PABPC3 | Myeloid | 0.63 | 0.20 | 2.62E-03 |
| PAK2 | Myeloid | 0.64 | 0.24 | 9.42E-03 |
| PBX1 | Platelets | 0.62 | 0.22 | 5.96E-03 |
| PCDH9 | B-cells | -0.67 | 0.19 | 1.15E-03 |
| PCF11 | GCSF down | -0.73 | 0.24 | 3.17E-03 |
| PCID2 | GCSF down | -0.54 | 0.20 | 8.65E-03 |
| PDCL3 | GCSF down | -0.58 | 0.20 | 5.38E-03 |
| PF4 | Platelets | 0.56 | 0.20 | 7.26E-03 |
| PF4V1 | Platelets | 0.69 | 0.21 | 1.49E-03 |
| PHF17 | GCSF down | -0.61 | 0.19 | 2.44E-03 |
| PJA1 | GCSF down | -0.57 | 0.20 | 6.55E-03 |
| PKNOX1 | Myeloid | -0.55 | 0.20 | 6.70E-03 |
| PLA2G12A | Platelets | -0.66 | 0.20 | 1.73E-03 |
| PLA2G7 | Platelets | 0.57 | 0.20 | 6.39E-03 |
| PLCL1 | GCSF down | -0.58 | 0.20 | 6.25E-03 |
| PLEKHA4 | B-cells | -0.56 | 0.21 | 8.83E-03 |
| PNKD | Platelets | 0.56 | 0.20 | 7.22E-03 |
| POFUT1 | GCSF down | 0.66 | 0.20 | 1.56E-03 |
| POLR3A | GCSF down | -0.57 | 0.21 | 7.95E-03 |
| POU2AF1 | B-cells | -0.61 | 0.20 | 4.45E-03 |
| PPIL1 | GCSF down | -0.66 | 0.21 | 2.16E-03 |
| PRCD | B-cells | -0.59 | 0.20 | 4.46E-03 |
| PRKAR2B | Platelets | 0.62 | 0.19 | 2.09E-03 |
| PSMA8 | GCSF down | -0.71 | 0.20 | 7.16E-04 |
| PTCD2 | GCSF down | -0.61 | 0.20 | 4.07E-03 |
| PTGIR | Platelets | 0.74 | 0.19 | 2.90E-04 |
| PTPLAD1 | GCSF down | -0.59 | 0.21 | 6.19E-03 |
| PTPN18 | Platelets | 0.59 | 0.21 | 7.31E-03 |
| PUS7 | B-cells | -0.57 | 0.21 | 8.67E-03 |
| QSOX2 | B-cells | -0.60 | 0.20 | 4.93E-03 |
| RASGRP3 | B-cells | -0.55 | 0.20 | 7.47E-03 |
| RECQL | GCSF down | -0.57 | 0.21 | 9.64E-03 |
| RFC4 | GCSF down | -0.55 | 0.20 | 7.34E-03 |
| RFESD | GCSF down | -0.68 | 0.20 | 1.28E-03 |
| RPL34 | GCSF down | -0.62 | 0.22 | 7.12E-03 |
| RPL39 | GCSF down | -0.63 | 0.21 | 3.81E-03 |
| RPP40 | GCSF down | -0.57 | 0.20 | 5.57E-03 |
| RRAS2 | GCSF down | -0.54 | 0.20 | 8.88E-03 |
| RUFY1 | Platelets | 0.56 | 0.20 | 7.58E-03 |
| SCCPDH | Platelets | 0.59 | 0.20 | 4.37E-03 |
| SCN1B | Platelets | 0.60 | 0.20 | 3.31E-03 |
| SCN3A | B-cells | -0.63 | 0.20 | 3.24E-03 |
| SEL1L3 | B-cells | -0.62 | 0.20 | 2.44E-03 |
| SESN1 | GCSF down | -0.61 | 0.21 | 5.84E-03 |
| SH3TC2 | Platelets | 0.66 | 0.19 | 1.22E-03 |
| SLC25A4 | GCSF down | -0.56 | 0.20 | 6.45E-03 |
| SLC25A42 | B-cells | -0.63 | 0.20 | 2.94E-03 |
| SLC35F2 | B-cells | -0.76 | 0.19 | 2.83E-04 |
| SLC48A1 | Platelets | 0.54 | 0.20 | 9.50E-03 |
| SMAGP | B-cells | -0.65 | 0.20 | 1.70E-03 |
| SNURF | GCSF down | -0.56 | 0.20 | 7.27E-03 |
| SNX1 | Myeloid | 0.60 | 0.20 | 4.68E-03 |
| SPAG16 | GCSF down | -0.57 | 0.20 | 6.31E-03 |
| SRP19 | GCSF down | -0.57 | 0.21 | 7.42E-03 |
| STAP1 | B-cells | -0.59 | 0.20 | 5.17E-03 |
| STIM2 | GCSF down | -0.55 | 0.20 | 8.57E-03 |
| STON2 | Platelets | 0.65 | 0.20 | 1.73E-03 |
| TAL1 | Platelets | 0.53 | 0.20 | 9.15E-03 |
| TARSL2 | GCSF down | -0.57 | 0.21 | 9.05E-03 |
| TAX1BP3 | Myeloid | 0.57 | 0.21 | 8.26E-03 |
| TBXA2R | Platelets | 0.56 | 0.20 | 7.01E-03 |
| TFE3 | Myeloid | 0.71 | 0.20 | 6.72E-04 |
| TIMELESS | B-cells | -0.60 | 0.21 | 5.92E-03 |
| TM4SF1 | Platelets | 0.64 | 0.20 | 2.35E-03 |
| TMEM11 | Myeloid | 0.61 | 0.21 | 5.38E-03 |
| TMEM156 | B-cells | -0.65 | 0.20 | 1.54E-03 |
| TPD52 | B-cells | -0.60 | 0.20 | 4.26E-03 |
| TRAM2 | GCSF down | -0.56 | 0.20 | 7.02E-03 |
| TRGC2 | GCSF down | -0.59 | 0.20 | 5.08E-03 |
| TTYH3 | Platelets | 0.70 | 0.19 | 7.04E-04 |
| TUBA1A | Myeloid | 0.59 | 0.20 | 5.49E-03 |
| TUBA1C | Myeloid | 0.61 | 0.20 | 4.19E-03 |
| TUBB1 | Platelets | 0.61 | 0.20 | 4.54E-03 |
| UBC | Myeloid | 0.66 | 0.20 | 1.57E-03 |
| UBXN8 | GCSF down | -0.77 | 0.19 | 1.33E-04 |
| USP36 | GCSF down | -0.54 | 0.20 | 8.42E-03 |
| VCAN | Myeloid | 0.55 | 0.21 | 9.95E-03 |
| VCL | Platelets | 0.55 | 0.20 | 9.43E-03 |
| VIM | Myeloid | 0.60 | 0.21 | 5.71E-03 |
| WDR52 | GCSF down | -0.63 | 0.19 | 1.90E-03 |
| WDYHV1 | GCSF down | -0.63 | 0.21 | 3.67E-03 |
| WFS1 | B-cells | -0.60 | 0.20 | 3.14E-03 |
| WHAMML2 | Platelets | 0.60 | 0.20 | 4.76E-03 |
| XRCC6BP1 | GCSF down | -0.62 | 0.20 | 2.54E-03 |
| ZDHHC13 | GCSF down | -0.56 | 0.20 | 8.21E-03 |
| ZDHHC23 | B-cells | -0.62 | 0.20 | 3.31E-03 |
| ZDHHC7 | Platelets | 0.72 | 0.19 | 4.89E-04 |
| ZNF135 | B-cells | -0.72 | 0.19 | 3.76E-04 |
| ZNF14 | GCSF down | -0.59 | 0.21 | 5.81E-03 |
| ZNF256 | B-cells | -0.64 | 0.20 | 2.66E-03 |
| ZNF391 | GCSF down | -0.68 | 0.20 | 1.24E-03 |
| ZNF439 | GCSF down | -0.81 | 0.20 | 1.78E-04 |
| ZNF525 | GCSF down | -0.70 | 0.20 | 8.81E-04 |
| ZNF564 | GCSF down | -0.56 | 0.20 | 5.92E-03 |
| ZNF607 | B-cells | -0.56 | 0.20 | 7.41E-03 |
| ZNF626 | GCSF down | -0.81 | 0.21 | 2.18E-04 |
| ZNF667 | B-cells | -0.57 | 0.20 | 7.51E-03 |
| ZNF670 | GCSF down | -0.55 | 0.20 | 7.68E-03 |
| ZNF673 | GCSF down | -0.68 | 0.21 | 1.85E-03 |
| ZNF80 | GCSF down | -0.57 | 0.21 | 7.31E-03 |
| ZNF891 | GCSF down | -0.62 | 0.20 | 3.00E-03 |
| ZNHIT3 | GCSF down | -0.66 | 0.21 | 3.00E-03 |
